# Myocardial Fibrosis Induced by Exposure to Subclinical Lipopolysaccharide Is Associated with Decreased miR-29c and Enhanced NOX2 Expression in Mice

**DOI:** 10.1371/journal.pone.0107556

**Published:** 2014-09-18

**Authors:** Wilbur Y. W. Lew, Evelyn Bayna, Erminia Dalle Molle, Riccardo Contu, Gianluigi Condorelli, Tong Tang

**Affiliations:** 1 Cardiology Section, Department of Medicine and Research Service, Veterans Administration San Diego Healthcare System, San Diego, California, United States of America; 2 University of California San Diego, San Diego, California, United States of America; 3 Humanitas Research Hospital, University of Milan, Rozzano, Milan, Italy; Albert Einstein College of Medicine, United States of America

## Abstract

**Background:**

Exposure to subclinical levels of lipopolysaccharide (LPS) occurs commonly and is seemingly well tolerated. However, recurrent LPS exposure induces cardiac fibrosis over 2 to 3 months in a murine model, not mediated by the renin-angiotensin system. Subclinical LPS induces cardiac fibrosis by unique mechanisms.

**Methods:**

In C57/Bl6 mice, LPS (10 mg/kg) or saline (control) were injected intraperitoneally once a week for 1–4 weeks. Mice showed no signs of distress, change in activity, appetite, or weight loss. Mice were euthanized after 3 days, 1, 2, or 4 weeks to measure cardiac expression of fibrosis-related genes and potential mediators (measured by QRT-PCR), including micro-RNA (miR) and NADPH oxidase (NOX). Collagen fraction area of the left ventricle was measured with picrosirius red staining. Cardiac fibroblasts isolated from adult mouse hearts were incubated with 0, 0.1, 1.0 or 10 ng/ml LPS for 48 hours.

**Results:**

Cardiac miR expression profiling demonstrated decreased miR-29c after 3 and 7 days following LPS, which were confirmed by QRT-PCR. The earliest changes in fibrosis-related genes and mediators that occurred 3 days after LPS were increased cardiac expression of TIMP-1 and NOX-2 (but not of NOX-4). This persisted at 1 and 2 weeks, with additional increases in collagen Iα1, collagen IIIα1, MMP2, MMP9, TIMP1, TIMP2, and periostin. There was no change in TGF-β or connective tissue growth factor. Collagen fraction area of the left ventricle increased after 2 and 4 weeks of LPS. LPS decreased miR-29c and increased NOX-2 in isolated cardiac fibroblasts.

**Conclusions:**

Recurrent exposure to subclinical LPS induces cardiac fibrosis after 2–4 weeks. Early changes 3 days after LPS were decreased miR-29c and increased NOX2 and TIMP1, which persisted at 1 and 2 weeks, along with widespread activation of fibrosis-related genes. Decreased miR-29c and increased NOX2, which induce cardiac fibrosis in other conditions, may uniquely mediate LPS-induced cardiac fibrosis.

## Introduction

Lipopolysaccharide (LPS) may circulate in the blood at subclinical levels and be a risk factor for cardiovascular disease. [1], [2] Circulating LPS from bacteria entering from the gut causes metabolic endotoxemia, with low grade inflammation, insulin resistance and weight gain. Increased circulating LPS occurs in healthy subjects after ingesting high fatty meals, [3], [4] in smokers, [2] with periodontal disease, [5] the metabolic syndrome, [1] heart failure, [6] diabetes mellitus, [7] liver disease, [1] or chronic infections of the lungs, gastrointestinal or urinary tract [2].

Acute exposure to subclinical LPS is well tolerated and seemingly benign. However, recurrent exposure to subclinical LPS induces cardiac fibrosis and increases mortality after 2–3 months in a murine model. 8] These adverse effects develop insidiously with no change in activity, appetite, weight, blood chemistries, left ventricular size or systolic function [8].

This may be important since exposure to subclinical LPS is common and cardiac fibrosis increases left ventricular stiffness, which is associated with heart failure with preserved ejection fraction (HFpEF). [9] HFpEF is as common as heart failure with reduced ejection fraction and is associated with significant morbidity and mortality, [10] but with few effective therapies [11].

The goals of this study were to examine mechanisms for subclinical LPS to induce cardiac fibrosis. Interestingly, common mediators of cardiac fibrosis in pathological remodeling including the renin-angiotensin system (RAS), transforming growth factor-β (TGF-β and TNF-α, [12] do not appear to be involved in cardiac fibrosis with subclinical LPS. [8] Therefore, novel mediators of fibrosis were considered, including microRNAs (miRs) and NADPH oxidase (NOX).

MicroRNAs are small, noncoding RNAs that regulate gene expression, including stress responses in the cardiovascular system. [13], [14] Amongst the hundreds of miRs, cardiac fibrosis has been associated with downregulation of miR-29, miR-30, miR-101, and miR-133 families, and with upregulation of miR-21. [15]–[17] LPS decreases miR-29 in liver fibrosis. [18], [19] It was hypothesized that LPS decreases miRs in the heart to induce cardiac fibrosis.

The NOX system is a major source of reactive oxygen species (ROS) in the heart, which may cause cardiac fibrosis. [20], [21] LPS activates the NOX system to generate ROS in macrophages, [22] peripheral blood mononuclear cells, [23] and in endothelial cells to cause vascular inflammation in acute lung injury. [24] It was postulated that LPS may activate NOX in the heart to contribute to cardiac fibrosis.

The major findings of this study are that subclinical LPS exposure decreases cardiac expression of miR-29c and increases NOX2 after 3 days, with subsequent activation of fibrotic factors that lead to cardiac fibrosis after 2–4 weeks of recurrent exposure to LPS. Since decreased miR-29c [25] and increased NOX2 [20], [26]–[28] precede activation of other fibrotic factors and are associated with cardiac fibrosis in other conditions, this suggests a unique role of miR-29c and NOX2 in LPS-induced cardiac fibrosis.

## Materials and Methods

### Ethics Statement

Experiments were performed in accordance with institutional guidelines and the “Guide for the Care and Use of Laboratory Animals”, Eighth Edition published in 2011. The studies were approved by the Institutional Animal Care and Use Committee and Research and Development Committee of the VA San Diego Healthcare System (Protocol #A11-007).

### Experimental Protocols

All methods have been described recently. [8] In brief, studies were performed in male C57Bl/6 mice weighing 25–30 g (Charles River Laboratories). Mice were injected i.p. with LPS (*Escherichia coli* 055, LPS no. B5, List Biological Laboratories, Campbell, CA) at a dose of 10 mg/kg (volume diluted with saline in 0.5 ml) once a week for 1–4 weeks. Mice were injected with 0.5 ml of saline as a control. Mice were monitored daily (7 days/week) for signs of distress, lethargy, labored breathing, anorexia, decreased appetite or fluid intake, or weight loss of ≥1–2 g over 2–3 days. Mice were euthanized if any of these signs or symptoms developed. At the end of the protocol, mice were euthanized by inducing deep anesthesia (with loss of paw withdrawal) using an i.p. injection of ketamine (100 mg/kg) and xylazine (10 mg/kg). The chest was opened and the heart excised surgically to induce death by exsanguination.

Cardiac fibrosis was assessed by imaging formalin-fixed, paraffin embedded left ventricular (LV) sections stained with picrosirius red, prepared as described previously. [8] The fractional area of fibrosis was quantified using NIH image software (ImageJ). LV sections were immunostained with antibodies to α-SMA and Ki67 to identify myofibroblasts and proliferating fibroblasts, as previously described [29].

Cardiac expression of fibrosis-related genes, inflammatory cytokines, and potential mediators of fibrosis were measured using quantitative reverse transcriptase-polymerase chain reaction (QRT-PCR). Total RNA extracted from the left ventricle was digested with RNase-free DNase, and reverse transcribed. QRT-PCR was performed and RNA equivalents normalized to simultaneously determined glyceraldehyde-3-phosphate dehydrogenase (GAPDH) mRNA levels in each sample.

Cardiac miR expression profiling was performed by a commercial source (LC Sciences, Houston, Texas) for mRNA prepared from LV samples.

Cardiac fibroblasts were isolated from adult mouse hearts as previously described [8]. In brief, hearts were suspended on a Langendorf apparatus and digested with a collagenase solution. Fibroblasts were plated in media, grown to 90–100% confluence and studied within two passages. Fibroblasts were starved and treated with 0.5% irradiated, low endotoxin FBS and incubated with 0, 0.1, 1.0 or 10 ng/ml LPS for 48 hours.

Statistical analysis was performed using SigmaPlot 11 (Systat Software, Inc. San Jose, CA), with multiple groups compared by two-way repeated measures analysis of variance (ANOVA). Multiple comparisons between two or more groups were made using the Holm-Sidak Test. Differences were considered to be significant at P<0.05, with additional tests used for post-hoc analyses as noted. Data are presented with mean and standard error of mean (SEM).

## Results

C57Bl/6 mice were injected with LPS (10 mg/kg) or saline i.p. once a week for up to four weeks. This dose of LPS was well tolerated with no signs of distress, anxiety or weight loss, as shown previously [8].

### Exposure to subclinical LPS induces cardiac fibrosis in 2–4 weeks

The time course for developing cardiac fibrosis was examined in four groups of C57Bl/6 mice injected with i.p. saline (control) or LPS 10 mg/kg/week for 1, 2, or 4 weeks. This dose of LPS was well tolerated with no signs of distress, anxiety or weight loss, as shown previously. [8] The heart was harvested 6–7 days after the last injection.

There was no evidence of cardiac hypertrophy, with no significant difference in LV weight normalized to body weight between any of the groups. Data for the combined right and left ventricle were similar, with no evidence of hypertrophy at any time period. This is similar to prior results showing no evidence of cardiac hypertrophy after 12–15 weeks of LPS [8].

**Figure 1:**
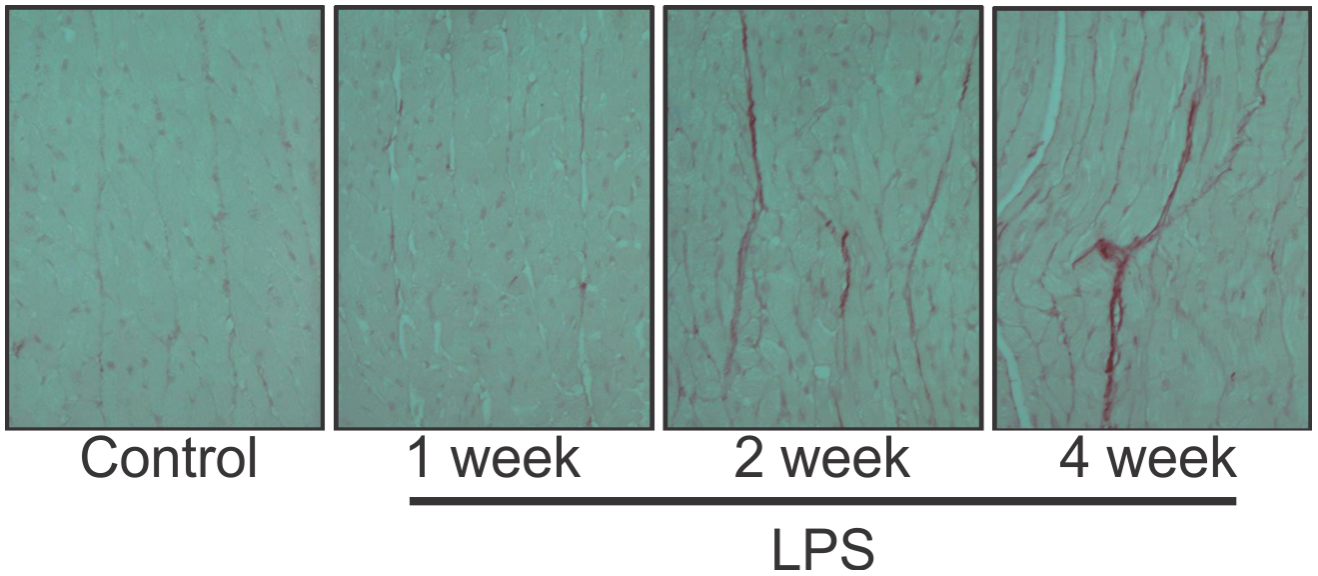
Cardiac fibrosis induced by weekly LPS. Picrosirius staining in the left ventricular wall increases after 2 or 4 weeks of intraperitoneal LPS (10 mg/kg/week), compared with a saline control mouse.

Figure 1 shows a representative example of photomicrographs of picrosirius red-stained sections from the left ventricle at the mid-ventricular level after i.p. saline (control), 1, 2, or 4 weeks of LPS (10 mg/kg/week). The fibrosis was mostly interstitial and occasionally perivascular with no obvious transmural differences, although this was not systematically examined.

**Figure 2:**
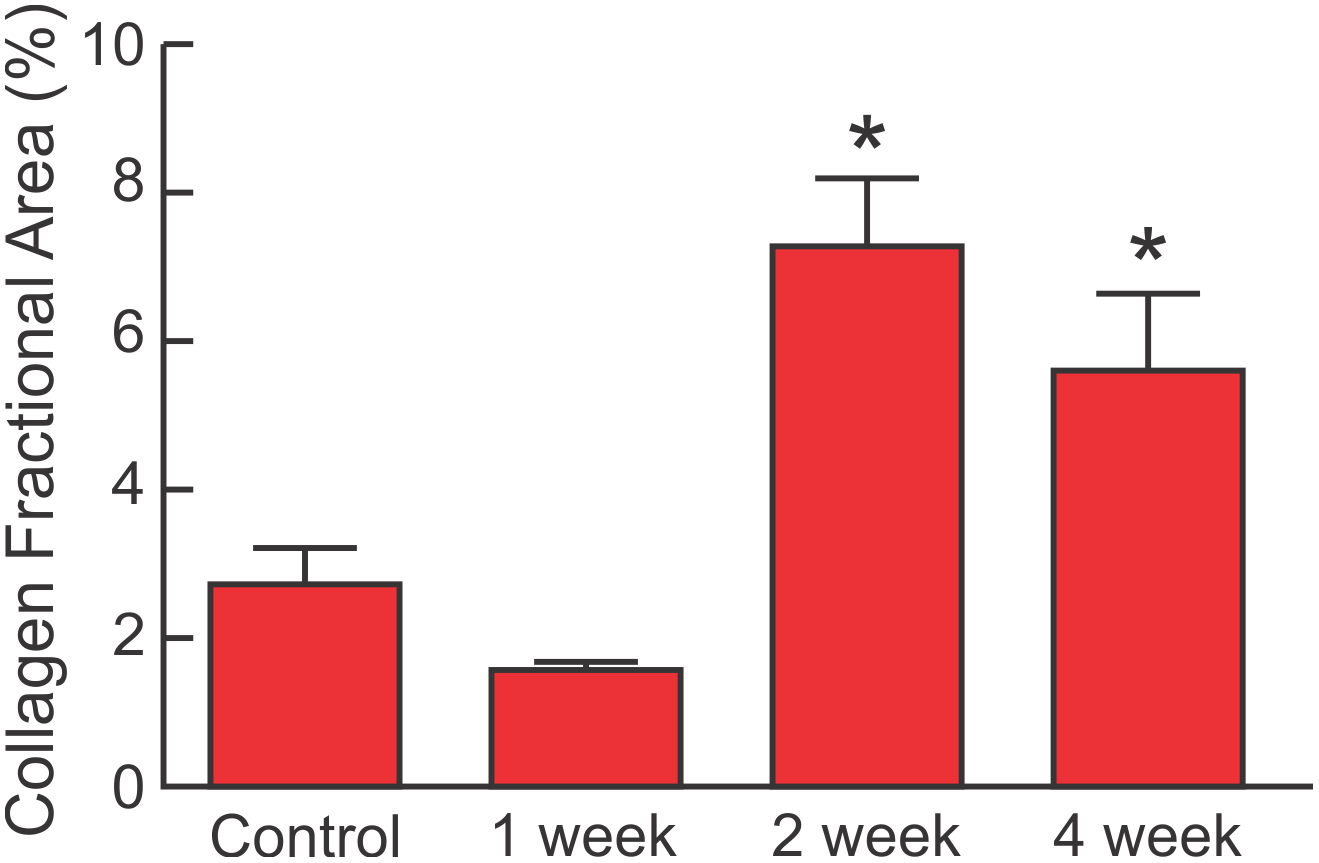
LPS increases collagen fraction area in 2–4 weeks. Collagen fraction area expressed as percent of left ventricle sections stained positive with picrosirius acid (mean + SEM) for mice injected with i.p. saline (Control, n = 7), or LPS 10 mg/kg/week for 1 week (n = 6), 2 weeks (n = 6), or 4 weeks (n = 5). There was a significant increase in collagen fraction area after injection of LPS (P<0.001, one way ANOVA). Multiple comparisons using the Holm-Sidak Test showed significant increases in collagen fraction area at 2 weeks and 4 weeks compared with Control or 1 week (*P<0.05), with no significant difference between 2 week and 4 weeks, or between Control and 1 week (P = ns).

Figure 2 shows collagen fraction area in the left ventricle (LV), measured by pircrosirius staining increased after 2 and 4 weeks of LPS, compared with control or 1 week after LPS. This was similar to the increase in LV collagen fraction area observed after 3 months of weekly LPS [8].

### Time course for subclinical LPS to activate fibrosis related genes and mediators

**Figure 3:**
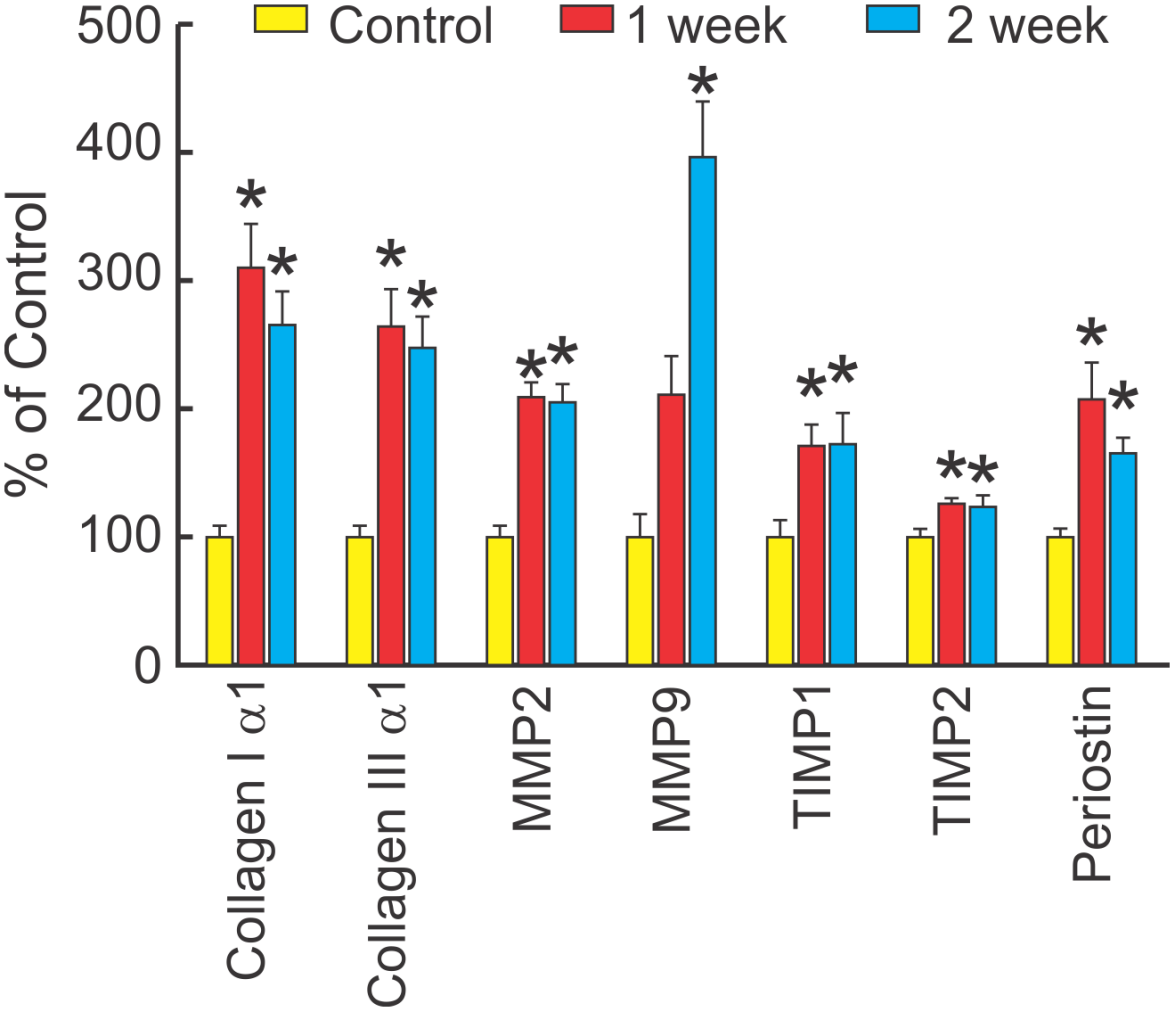
LPS increases fibrosis-related genes in 1–2 weeks. Cardiac expression of fibrosis-related genes (mean + SEM) measured by QRT-PCR in mice injected with i.p. saline (Control, n = 7), or LPS 10 mg/kg/week for 1 week (n = 6) or for 2 weeks (n = 6). LPS significantly increased collagen Iα1, collagen IIIα1, MMP2, MMP9, TIMP1, TIMP2, and periostin in the left ventricle (P<0.001 for all, except P = 0.012 for TIMP1, one way ANOVA). Multiple comparisons (Holm-Sidak Test) showed significant increases in all fibrosis related genes at 1 week or 2 weeks compared with control (*P<0.05 for all, except P = ns for MMP9 at 1 week). There were no significant differences in fibrosis related genes between 1 week and 2 weeks, except for a significant increase in MMP9 (P<0.05).

The time course for activation of fibrosis-related genes was evaluated in three groups of mice injected with i.p. saline (control) or LPS 10 mg/kg/week for 1 or 2 weeks. Figure 3 shows that LPS increased cardiac expression of several fibrosis-related genes one week after LPS compared with control, including collagen Iα1, collagen IIIα1, MMP2, TIMP1, TIMP2, and periostin. These changes remained comparable to the increase observed after 2 weeks of LPS. In addition, there was an increase in MMP9 after 2 weeks of LPS compared with 1 week or control. There was no significant change in TIMP3 or TIMP4 at any time (not shown). These changes were similar to the profile of increased cardiac expression of collagen Iα1, collagen IIIα1, MMP2, MMP9, TIMP1, and periostin observed after 3 months of exposure to weekly LPS [8].

**Figure 4:**
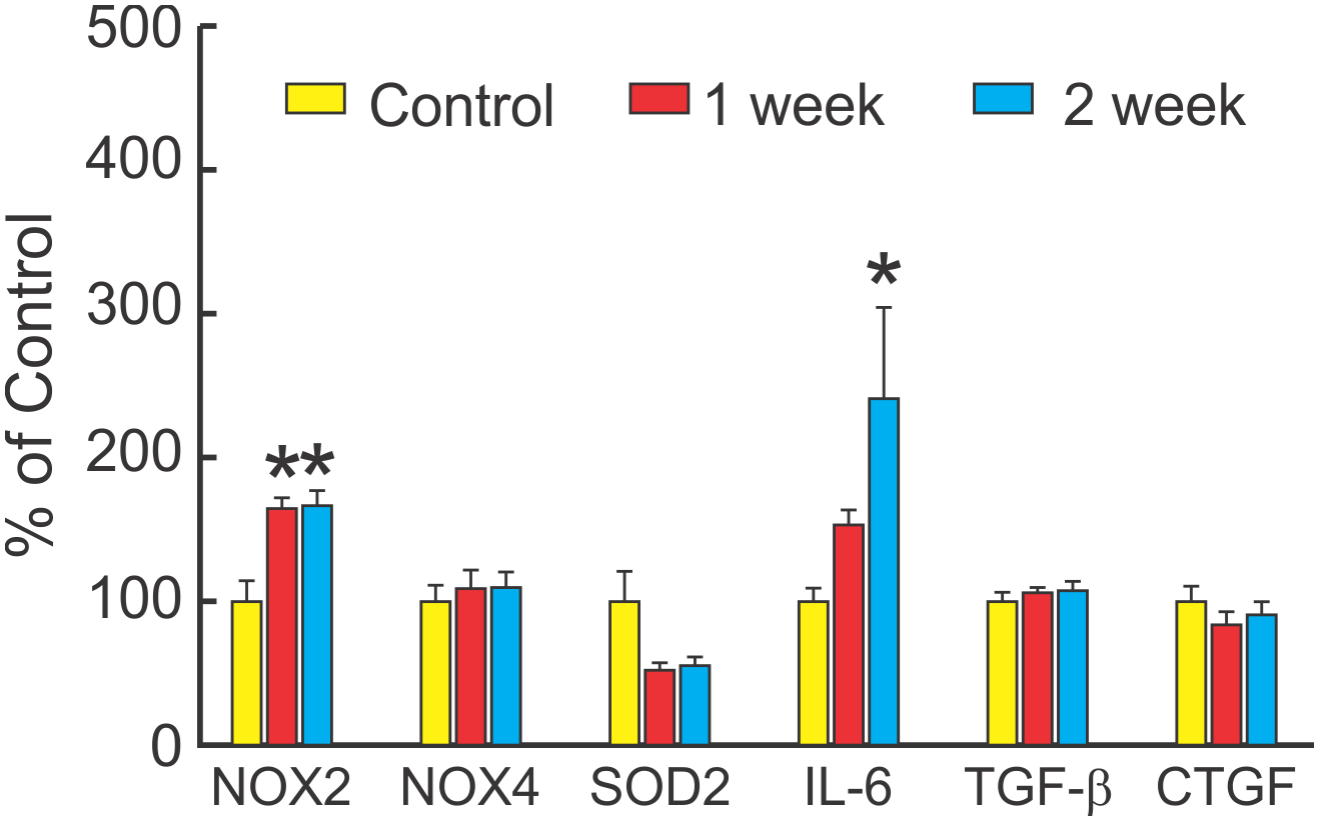
LPS increases NOX2 in 1–2 weeks. Cardiac expression of mediators of fibrosis (mean + SEM) measured by QRT-PCR in mice injected with i.p. saline (Control, n = 7), or LPS 10 mg/kg/week for 1 week (n = 6) or for 2 weeks (n = 6). LPS significantly increased NOX2 (P = 0.002) and IL-6 (P = 0.039, one way ANOVA), but not NOX4, transforming growth factor- β (TGF-β) or connective tissue growth factor (CTGF) (all P = ns). There was a tendency for LPS to decrease SOD2 (P = 0.057). Multiple comparisons (Holm-Sidak Test) showed that compared with control, there were significant increases in NOX2 at 1 week or 2 weeks and IL-6 at 2 weeks (*P<0.05), with no difference between 1 or 2 weeks.

Potential mediators of fibrosis were evaluated from the same mice. Figure 4 shows that compared with control, there was a significant increase in cardiac expression of NOX2 at 1 and 2 weeks after LPS (with no difference between the two time periods). There was no change in NOX4. There was a trend for decreased expression of SOD2. Cardiac expression of IL-6 increased after 2 weeks of LPS.

There was no change in TGF-β or connective tissue growth factor (CTGF) at any time, which are important mediators of cardiac fibrosis in other conditions. The prior study showed no significant change in TGF-β or TNF-α after 3 months of weekly LPS [8].

**Figure 5:**
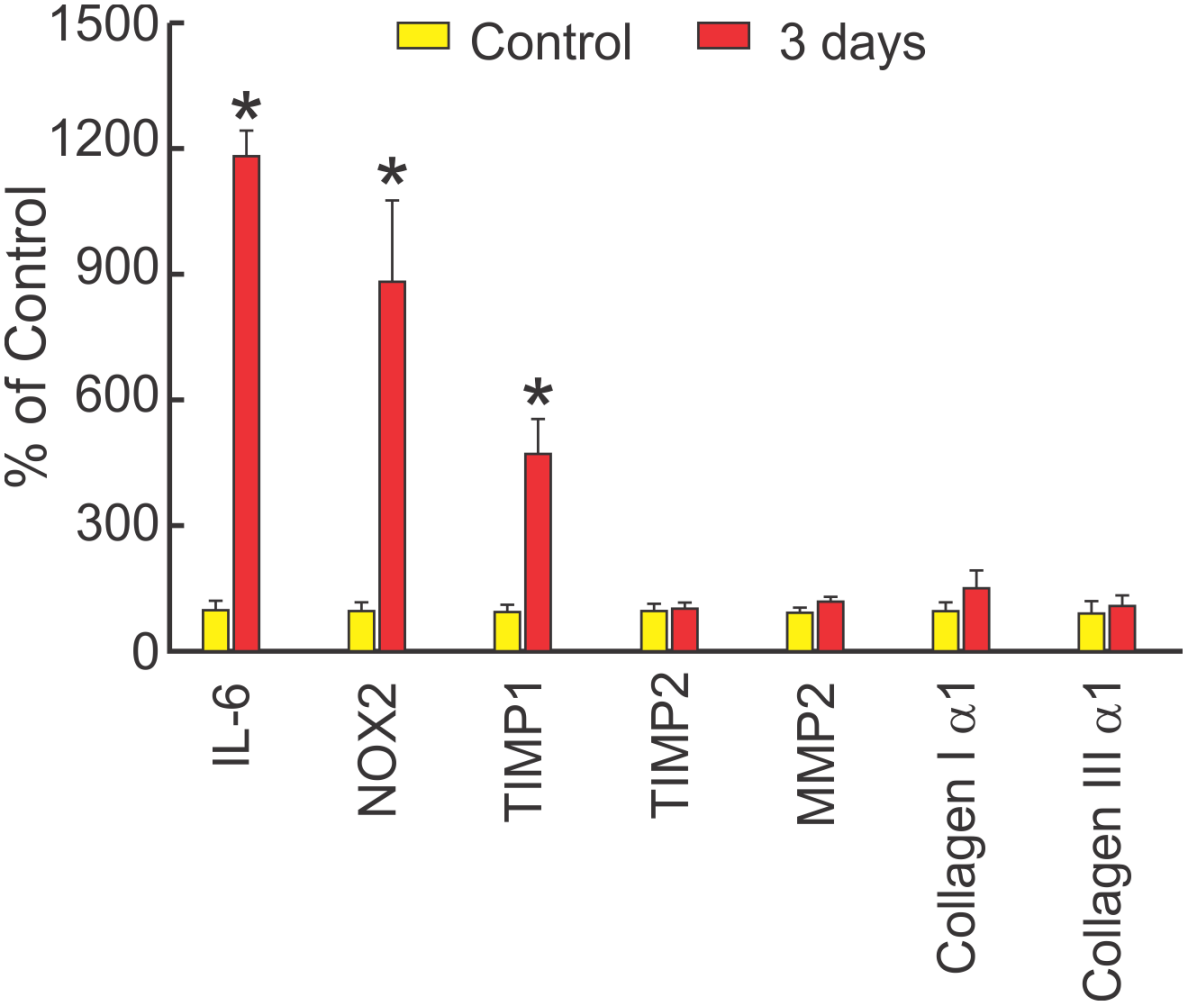
LPS increases NOX2, TIMP1 and IL-6 in 3 days. Cardiac expression of fibrosis-related genes (mean + SEM) in mice after an i.p. injection of saline (Control, n = 3), or three days after LPS 10 mg/kg (n = 3). LPS significantly increased IL-6, NOX2, and TIMP1 (*P<0.05), with no significant change in TIMP2, MMP2, collagen I α1, or collagen III α1.

To assess which profibrotic factors are activated early after LPS, measurements were made in two groups of mice three days after i.p. injection of LPS (10 mg/kg) or saline (control). Figure 5 shows that at 3 days, LPS increased cardiac expression of IL-6, NOX2, and TIMP1 compared with control, but not TIMP2, MMP2, collagen Iα1, nor collagen IIIα1.

**Figure 6:**
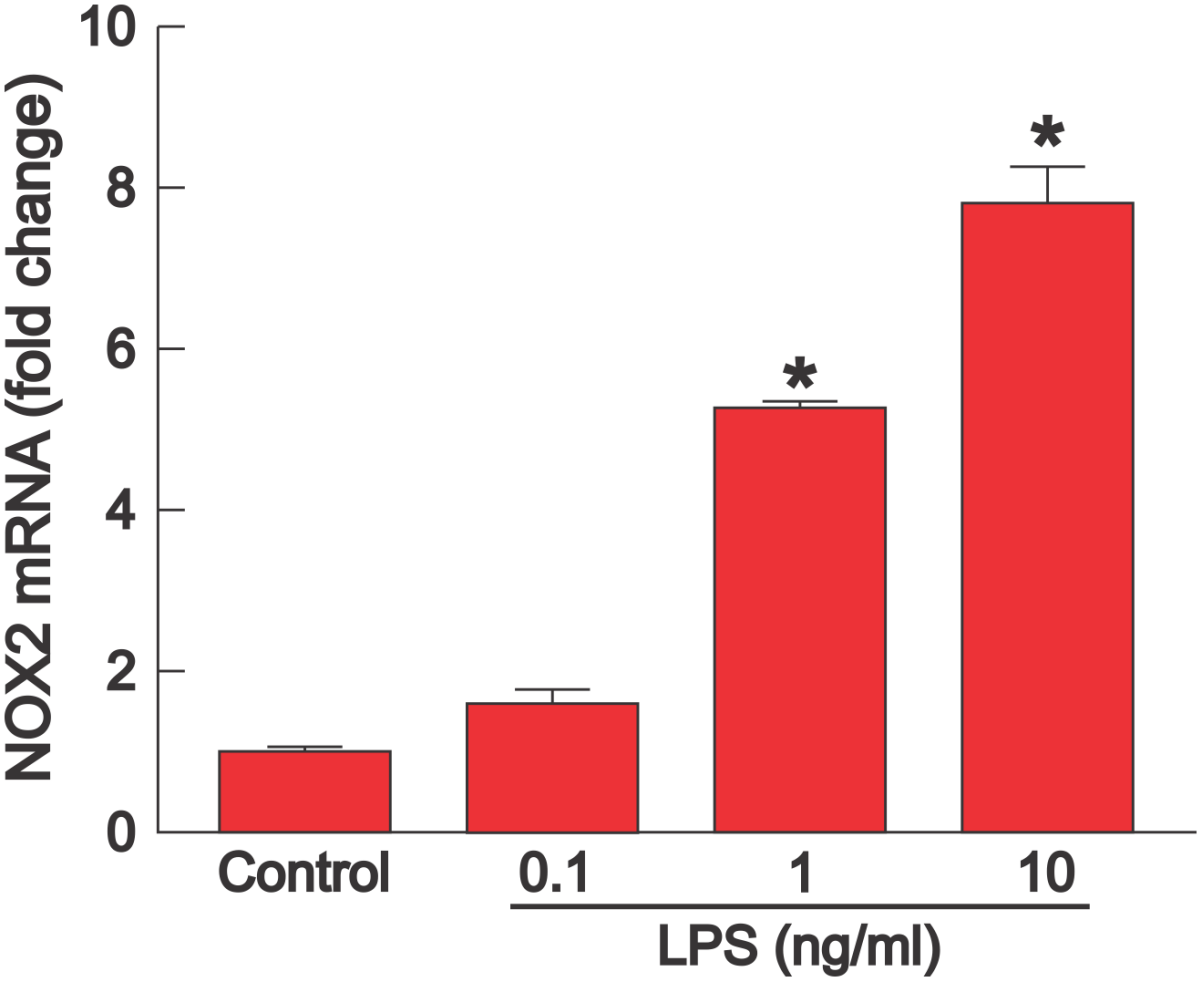
LPS increases expression of NOX2 in isolated cardiac fibroblasts. Expression of NOX2 mRNA (mean + SEM, n = 3) was measured in isolated cardiac fibroblasts from adult mice 48 hours after incubation with 0 (control), 0.1, 1, or 10 ng/ml LPS. Expression of NOX2 increased significantly with LPS doses 1 or 10 ng/ml compared with LPS 0.1 ng/ml or control (P<0.05). Expresssion of NOX2 mRNA was higher with 10 than 1 ng/ml LPS (P<0.05).

To assess if LPS targets cardiac fibroblasts, fibroblasts isolated from adult mouse hearts were incubated with LPS for 48 hours. Figure 6 demonstrates that LPS produced a dose-dependent increase in NOX2 mRNA expression, which was significantly higher with 1 or 10 ng/ml LPS compared with control or 0.1 ng/ml LPS (P<0.05). There was a greater increase in NOX2 with 10 than 1 ng/ml LPS (P<0.05); there was no difference between 0.1 ng/ml LPS and control.

### Cardiac miR expression profile with subclinical LPS

**Figure 7:**
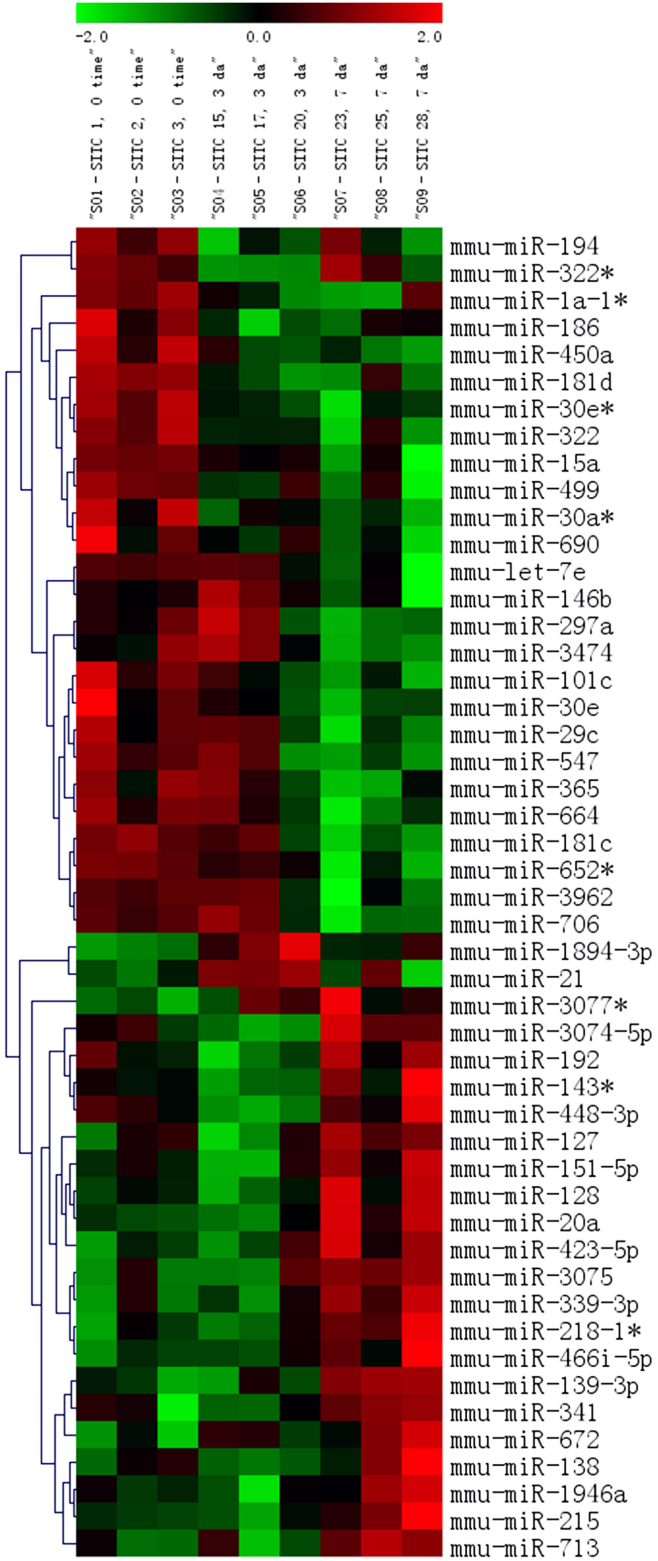
Cardiac miRNA expression profiling after LPS. Cardiac miRNA expression profiling in C57/Bl6 mice injected with i.p. saline (time 0, control), or with hearts harvested 3 or 7 days after LPS (10 mg/kg) (n = 3 mice each time period). Heat map shows miR expression that increased (red), did not change (black) or decreased (green) for three mice each at time 0 (control, columns 1–3), 3 days after LPS (columns 4–6), and 7 days after LPS (columns 7–9), with differences between the three time periods with a p-value of<0.10 (ANOVA).

To assess if dysregulation of miRs occurs with LPS-induced cardiac fibrosis, miR expression profiling was performed in left ventricular samples from three groups of mice injected with i.p. saline (control) or LPS (10 mg/kg), with hearts harvested 3 or 7 days later. Figure 7 shows a heat map with miR expression that increased (red), did not change (black), or decreased (green) for each mouse at time 0 (control), 3, or 7 days after LPS.

Cardiac fibrosis has been associated with decreases in miR-29, [25] miR-133, miR-30, [30] miR-101 [17] and/or increased miR-21 [31], [32] in pathological conditions (e.g. ischemia-reperfusion, hypertrophy and heart failure). The intensities for several of these miRs did not change over 3–7 days, including miR-29a, miR-29b, miR-30, miR-101 or miR133 families. There was a transient elevation in miR-21 on day 3 that returned to baseline by day 7.

The most consistent change was a decrease in miR-29c: intensity at baseline (on day 0) was 2082±580 and this decreased on day 3 to 1552±365, and on day 7 to 613±165 after LPS (P = 0.05, ANOVA).

**Figure 8:**
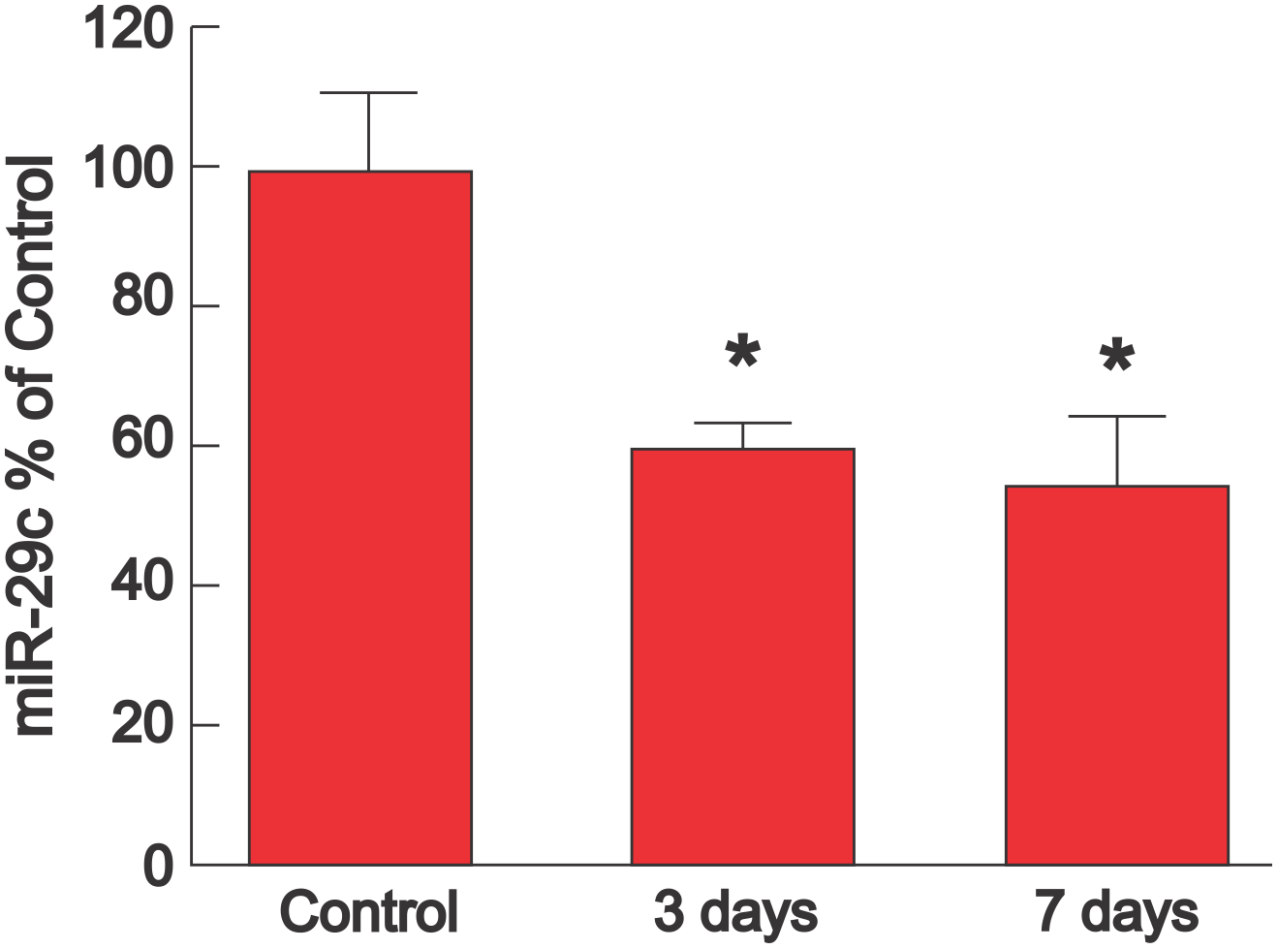
LPS decreases cardiac expression of miR-29c for 3–7 days. Cardiac expression of miR-29c (mean + SEM) measured by QRT-PCR in mice injected with i.p. saline (time 0, control, n = 3), 3 days after LPS (10 mg/kg i.p.) (n = 3), or 7 days after LPS (10 mg/kg i.p.) (n = 3). There was a significant decrease in miR-29c with LPS (P = 0.02, one way ANOVA). Multiple comparisons (Holm-Sidak Test) showed that when compared with control, the decrease in miR-29c was significant at 3 days and at 7 days (*P<0.05), with no difference between 3 days and 7 days.

To confirm this result, QRT-PCR was performed from LV tissue samples from the same protocol. Figure 8 shows that miR-29c was decreased on days 3 and 7 after LPS compared with control. There was no significant change in miR-21 (not shown).

**Figure 9:**
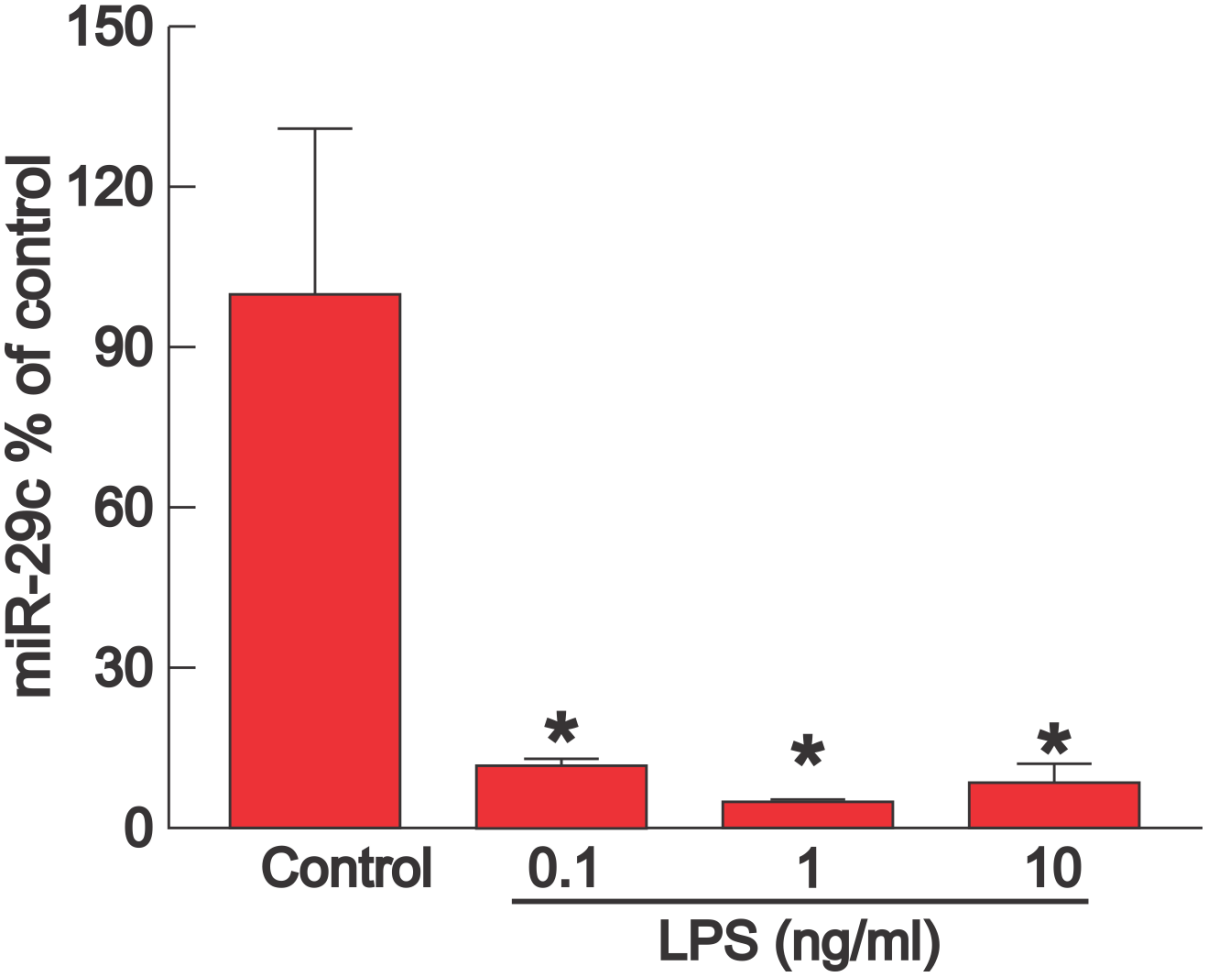
LPS decreases expression of miR-29c in isolated cardiac fibroblasts. Expression of miR-29c (mean + SEM, n = 3) was measured in isolated cardiac fibroblasts from adult mice 48 hours after incubation with 0 (control), 0.1, 1, or 10 ng/ml LPS. Each dose of LPS significantly decreased miR-29c compared with control (P<0.05), with no difference between LPS doses.

To assess if LPS decreases miR-29c in fibroblasts, cardiac fibroblasts isolated from adult mice were incubated with LPS for 48 hours. Figure 9 shows that LPS produced a marked decrease in miR-29c levels, which were significantly lower with 0.1, 1, or 10 ng/ml LPS compared with control (P<0.05). There was no difference between any of the LPS doses.

### Summary of temporal changes

Table 1 summarizes cardiac factors that increase, decrease, or remain unchanged at 3 days, and 1 and 2 weeks after weekly exposure to subclinical LPS, compared with control. The earliest changes that occurred 3 days after LPS were a decrease in miR-29c, and increases in IL-6, NOX2, and TIMP1 expression. The decrease in miR-29c and increases in NOX2 and TIMP1 persisted at 1 week, along with increases in additional fibrotic factors, including TIMP2, MMP2, collagen IαI, collagen IIIα1, and periostin. These changes persisted at 2 weeks, with an additional increase in MMP9 and evidence of fibrosis with increased collagen fraction area. Several of these changes persisted chronically after weekly exposure subclinical LPS for 2–3 months [8].

**Table 1:** Time course for changes in fibrosis related factors in the left ventricle after injection of LPS (10 mg/kg/week i.p.) compared with control (saline injection).

	3 days	1 week	2 weeks
**collagen fraction area**		no change	**Increase**
**miR-29c**	***Decrease***	***Decrease***	
**IL-6**	**Increase**	no change	**Increase**
**NOX2**	**Increase**	**Increase**	**Increase**
**TIMP1**	**Increase**	**Increase**	**Increase**
**TIMP2**	no change	**Increase**	**Increase**
**MMP2**	no change	**Increase**	**Increase**
**MMP9**		no change	**Increase**
**collagen I α1**	no change	**Increase**	**Increase**
**collagen III α1**	no change	**Increase**	**Increase**
**periostin**	no change	**Increase**	**Increase**

NOX 2 = NADPH Oxidase 2, MMP = matrix metaloproteinase, TIMP = tissue inhibitors of MMPs.

## Discussion

The major findings of this study are that exposure to subclinical LPS activates mediators in the heart within days, with activation of pro-fibrotic factors after one week leading to cardiac fibrosis after two weeks. The earliest changes are decreased cardiac expression of miR-29c with increased IL-6, NOX2, and TIMP1 three days after LPS. After one week, there was increased cardiac expression of collagen Iα1, collagen IIIα1, TIMP2, MMP2, and periostin, which persisted at two weeks. At two weeks there was an additional increase in MMP9 and cardiac fibrosis with increased collagen fraction area in the left ventricle.

Cardiac structure is maintained by a dynamic balance of extracellular matrix proteins, including MMPs and TIMPs. [33] The myofibroblast plays a central role in mediating signaling pathways that cause an imbalance between collagen synthesis and degradation resulting in cardiac fibrosis. [12] The profile of increased cardiac expression of collagen Iα1, collagen III α1, MMP2, MMP9, TIMP1, TIMP2, and periostin 2 weeks after LPS, was similar in magnitude to the changes observed after 2–3 months of weekly exposure to subclinical LPS [8].

Cardiac fibrosis is mediated by TGF-β and/or CTGF in several pathological conditions. [12] LPS did not change in mRNA expression of TGF-β or CTGF, but decreased miR-29c and increased NOX2 mRNA expression. This unique pattern of mRNA expression may reflect the insidious development of cardiac fibrosis due to subclinical LPS, in contrast to the fibrosis that develops in response to overt diseases, such as myocardial ischemia, heart failure, or hypertrophy. Although mRNA expression of TGF-β did not change, this does not exclude a potential role in mediating LPS-induced cardiac fibrosis since large amounts of latent TGF-β are present in normal hearts, which can be activated by a variety of stimuli (e.g. ROS generation) in the absence of any change in TGF-β expression [34].

The decrease in miR-29c and increase in NOX2 may be novel mediators of cardiac fibrosis induced by subclinical LPS. The miRs are small (approximately 22 nucleotides long), non-coding RNAs that regulate gene expression typically by binding to the 3′ untranslated region to inhibit translation and/or decrease stability to increase degradation. [13], [14], [35] There are several hundred miRs in mammals; each miR may have multiple target sites. This network allows small changes in miR levels to regulate several cardiovascular processes, including development, hypertrophy, angiogenesis, ion channel function, remodeling, and fibrosis. Individual miRs can be modulated as novel therapeutic targets in cardiovascular diseases [35], [36].

Cardiac fibrosis is associated with downregulation of miR-29, miR-30, miR-101, and miR-133, and upregulation of miR-21. [15]–[17] The cardiac fibrosis that develops with decreased miR-133 and miR-30c involves CTGF, [30] which did not change with LPS. There was no significant change in miR-133, miR-30, or miR-101 family members after LPS. There was a transient increase in miR-21 on day 3 by miR expression profiling, which was not significant when measured by QRT-PCR.

The most consistent change in fibrosis-related miRs was a significant decrease in miR-29c on miR expression profiling, which was confirmed by QRT-PCR, and persisted from 3 to 7 days after LPS. There was no significant change in miR-29a or miR-29b.

The miR-29 family is the fourth most abundant miR in the heart, [37] with preferential expression in fibroblasts. [38] The miR-29 family includes 3 members expressed from a bicistronic cluster with miR-29a coexpressed with miR-29b-1, and miR-29b-2 coexpressed with miR-29c. All three miR-29 family members are downregulated with myocardial ischemia-reperfusion in mice and humans, particularly in the border zone. [25] A number of fibrosis-related genes are targeted by miR-29, including collagens, fibrillins, and elastin to induce cardiac fibrosis. In vivo inhibition of miR-29 with an antagomir, an oligonucleotide complementary to miR-29b (anti-miR-29b), activates collagen expression [25].

Circulating miRs may be elevated in cardiac diseases and serve as biomarkers. In patients with hypertrophic cardiomyopathy, several miRs, including miR-29a, are elevated and correlate with ventricular remodeling and the degree of cardiac hypertrophy [39] Only miR-29a correlated with the extent of cardiac fibrosis, as measured by cardiac magnetic resonance [39].

miR-29 plays a role in fibrosis also in the liver. LPS from the gut exacerbates hepatic fibrosis after chemical injury. [18] Hepatic stellate cells, the major cell in hepatic fibrogenesis, are rapidly (1–2 hours) activated by LPS to decrease miR-29a, miR-29b, and miR-29c. [19] The gut is the source of subclinical LPS in metabolic endotoxemia. [1] It is unknown if LPS from the gut plays a similar role to decrease cardiac miR-29c in cardiac fibrosis. Subclinical LPS induced cardiac fibrosis without preceding myocardial injury, in contrast to exacerbation of hepatic fibrosis induced by chemical injuries [18].

The miR-29 family directly regulates at least 16 extracellular matrix genes. [40] Downregulation of miR-29 activates several extracellular matrix proteins that play important roles in cardiac fibrosis. [36] In the kidney, downregulation of miR-29c is associated with renal interstitial fibrosis with increased collagen type II α1 and tropomyosin 1α, which are attenuated by activating hypoxia-inducible factor-α (HIF-α). [41] Downregulation of miR-29c in fibroblasts activates extracellular matrix genes resulting in fibrotic extracellular changes in idiopathic pulmonary fibrosis. [42] Identifying the target sites and the role of miRs have generated considerable interest as manipulating miRs may be a novel therapeutic approach to treat cardiovascular diseases, including cardiac fibrosis [36], [43].

In the current study, low dose LPS markedly decreased miR-29c in isolated cardiac fibroblasts. Since several fibrosis-related genes are directly activated by decreased miR-29 [25], decreased miR-29c may play an important role in the LPS-induced cardiac fibrosis.

Subclinical LPS may induce oxidative stress with reactive oxygen species (ROS). The NOX system is a major source of ROS in the heart. [21], [44], [45] The NOX family contains 7 members, with NOX2 and NOX4 the predominant isoforms in the heart and expressed in cardiac myocytes, fibroblasts, and endothelial cells. LPS activates NOX4 to generate ROS in endothelial cells. [46] Angiotensin II or aldosterone increase NOX2 activity to activate profibrotic genes with interstitial fibrosis [26].

There are links between LPS responses and NOX. LPS increases NOX2- mediated ROS generation and MMP9 in macrophages, [22] or NOX4-mediated increases in H_2_O_2_ and IL-6 release in peripheral blood mononuclear cells. [23] In acute lung injury, LPS causes endothelial dysfunction with increased vascular permeability by NOX2- and ROS-mediated pathways. [24] In the current study, LPS increased cardiac NOX2, but not NOX4, after 3 days, which persisted after 1 and 2 weeks. In isolated cardiac fibroblasts, LPS dose-dependently increased NOX2 mRNA expression, which may be an important cellular target. It is possible that LPS activates NOX2 in other cells as well, which may play a potential role. Since increased NOX2 has been associated with cardiac fibrosis in other diseases, [20], [26]–[28] it may play a similar role in LPS-induced cardiac fibrosis.

The earliest mediators changes were an increase in IL-6 and NOX2, and a decrease in miR-29c. LPS dose-dependently activates isolated adult cardiac fibroblasts to increase IL-6 within 48 hours. [8] IL-6 can convert cardiac fibroblasts to myofibroblasts with collagen deposition. [47] In the current study, LPS decreased miR-29c and increased NOX2 in isolated adult mouse cardiac fibroblasts. Collectively, the results suggest that miR-29c and NOX2 may mediate LPS-induced cardiac fibrosis, with the cardiac fibroblast a key target for LPS. Additional studies are required to determine the role of each mediator, and how they may interact to induce cardiac fibrosis. Novel therapies that target miR-29c and/or NOX2 may be required to attenuate LPS-induced fibrosis. The development of new therapies to target miRs [35], [36] and NOX [48] have emerged as an active area of investigation.

The clinical significance of this study is that exposure to subclinical LPS is common (e.g. after high fatty meals or smoking, with metabolic endotoxemia or metabolic syndrome) with the potential adverse effect of inducing cardiac fibrosis. Cardiac fibrosis decreases cardiac compliance and contributes to heart failure with preserved ejection fraction (HFpEF). [9] Although HFpEF is similar to heart failure with reduced ejection fraction in terms of incidence and mortality, [10] in contrast, no therapies have been found to be effective for treating HFpEF. [11] If subclinical LPS contributes to HFpEF, this may provide novel targets to ameliorate this burgeoning problem.

## Conclusion

In conclusion, recurrent exposure to subclinical levels of LPS induces cardiac fibrosis after 2–4 weeks. This is related to a decrease in cardiac miR-29c and an increase in NOX2 expression, which are associated with cardiac fibrosis. Therapies targeting miR-29c and/or NOX2 may be required to attenuate LPS-induced cardiac fibrosis, and prevent excess mortality associated with recurrent exposure to subclinical LPS [8].
